# Hyperfunctioning parathyroid gland and skeletal involvement on [^18^F]fluorocholine PET/CT: one look with two views

**DOI:** 10.1186/s41824-022-00149-y

**Published:** 2022-12-12

**Authors:** Carmela Nappi, Leandra Piscopo, Michele Klain, Ciro Gabriele Mainolfi, Emilia Vergara, Daniela Adamo, Michele Davide Mignogna, Alberto Cuocolo

**Affiliations:** 1 Department of Advanced Biomedical Sciences, University of Naples Federico II, Napoli, Italy; 2 Department of Neuroscience, Reproductive Sciences and Dentistry, University of Naples Federico II, Napoli, Italy

## Abstract

Hyperparathyroidism is an endocrine disorder that may be associated with other metabolic diseases. Non-invasive imaging techniques including [^99m^Tc]Tc-sestamibi single-photon emission computed tomography (SPECT) and [^18^F]fluorocholine positron emission tomography (PET)/computed tomography (CT) play a key role on management of patients with hyperparathyroidism. We report for the first time a case of a patient with evidence of both hyperfunctioning parathyroid tissue and multiple lytic bone lesions on [^18^F]fluorocholine PET/CT imaging. The present case report highlights the potential role of whole-body [^18^F]fluorocholine PET/CT for the identification of both parathyroid adenoma and multiple bone lesions in a single diagnostic setting.

## Introduction

Hyperparathyroidism is an endocrine disorder (Evangelista et al. 2020). Primary hyperparathyroidism is related to autonomous overproduction of parathyroid hormone (PTH) by parathyroid glands. In most cases, the disease is caused by a single parathyroid adenoma (89%) and less commonly by parathyroid hyperplasia (6%), double parathyroid adenoma (4%), or parathyroid carcinoma (< 1%) (Younes et al. 2005). The secondary form of hyperparathyroidism is caused by a chronic stimulus on the parathyroid glands due to low circulating calcium levels, while in tertiary hyperparathyroidism, the parathyroid glands take on their own functional autonomy due to a continuous stimulus from secondary hyperparathyroidism. The classic symptoms of hyperparathyroidism are characterized by painful bones, kidney stones, fatigue overtones, hypercalcemia, abdominal cramps and in some cases mental disorders (Younes et al. 2005). Non-invasive imaging techniques play a key role in the management of hyperparathyroidism. Both [^99m^Tc]Tc-sestamibi single-photon emission tomography (SPECT) and [^18^F]fluorocholine positron emission tomography (PET)/computed tomography (CT) boast high sensitivity in the identification of hyperfunctioning parathyroid lesions (Petranović Ovčariček et al. 2021; Zhang et al. 2020; Broos et al. 2019). [^99m^Tc]Tc-sestamibi is endowed with a mitochondrial mechanism of action demonstrating high uptake in cells with a high mitotic index, of both benign and malignant nature (Evangelista et al. 2020; Assante et al. 2019).

It has been proved that [^18^F]fluorocholine plays an important role in the localization of prostate cancer lesions in patients with biochemical recurrence of the disease (Hodolic 2011; Schillaci et al. 2010). The rationale behind its use as oncological tracer is the high proliferative capacity of tumor cells, which will consequently contain a large amount of phosphatidylcholine and other phospholipids. Thus, choline is incorporated into cells in active proliferation with an increase in phosphorylcholine for the synthesis of cell membranes (Katz-Brull et al. 1998; Zeisel 1981). Recently, this tracer has been also considered a potential molecular probe for functional parathyroid evaluation (Petranović Ovčariček et al. 2021; Zhang et al. 2020; Broos et al. 2019).

While [^99m^Tc]Tc-sestamibi SPECT is the currently reference imaging for functional parathyroid evaluation, the high resolution of PET/CT, using [^18^F]fluorocholine as a radioactive tracer, allows to visualize very small parathyroid lesions with high morphological detail and incremental diagnostic value as compared to [^99m^Tc]Tc-sestamibi SPECT (Beheshti et al. 2018). Moreover, the whole-body scan helps in detecting ectopic foci of hyperfunctioning parathyroid glands.

Yet, hyperparathyroidism may be associated with other metabolic disorders (Dumitrescu and Collins 2008). It can be associated to fibrous dysplasia as also previously described by McCune (1936) and by Albright et al. (1937), Turcu and Clarke (2013) and Collins et al. (2012), or it can be associated with juvenile ossifying fibroma (Dominguete et al. 2008). We report a case of a patient with evidence, on [^18^F]fluorocholine PET/CT imaging, of both hyperfunctioning parathyroid tissue and multiple lytic bone lesions.

## Case report

A young woman of 26 years was admitted to Department of Neuroscience, Reproductive Sciences and Dentistry, University of Naples Federico II, due to a swelling in the left jawbone. An incisional biopsy of the left maxillary lesion was performed, which proved to be suggestive of fibrous-bone lesion due to certain characteristics, including the presence of fibrous struma, immature bone trabeculae and a high proportion of multinucleated giant cells, without atypia. Contextually, a series of blood tests showed an increase in serum levels of PTH (1320.0 pg/ml), alkaline phosphatase (379 U/L), calcium (15.8 mg/dl), a lowering of the vitamin D values (8.8 ng/mL) and phosphorous (2.1 mg/dl). Of note, further laboratory examinations and clinical workup excluded any suspicion of the presence of other malignancies characterized by bone lytic lesions.

**Fig. 1:**
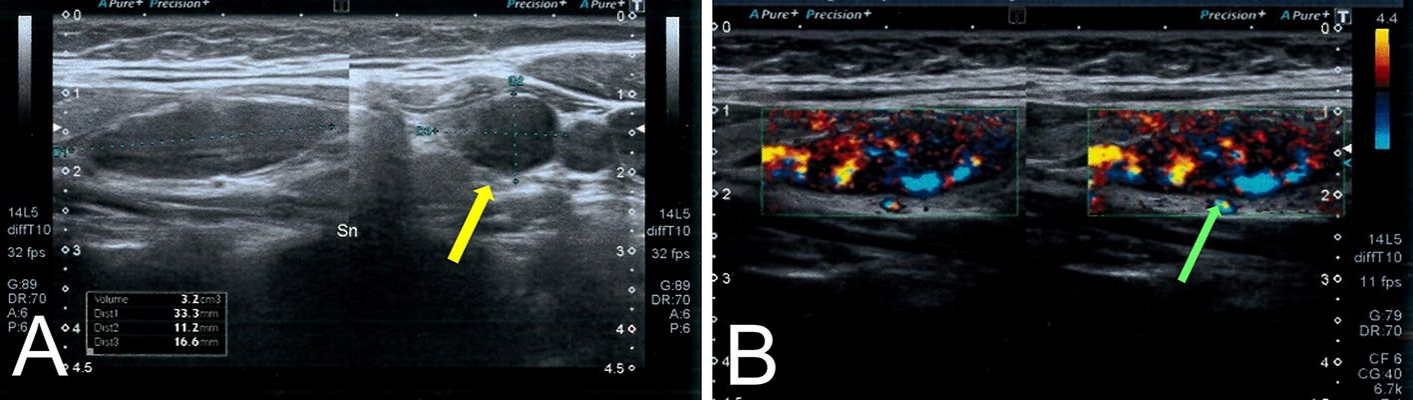
Ultrasound (**A**) showing hyperplastic parathyroid tissue in the lower left parathyroid lodge (yellow arrow) with contextual vascularization at color-power-Doppler (green arrow) (**B**)

**Fig. 2:**
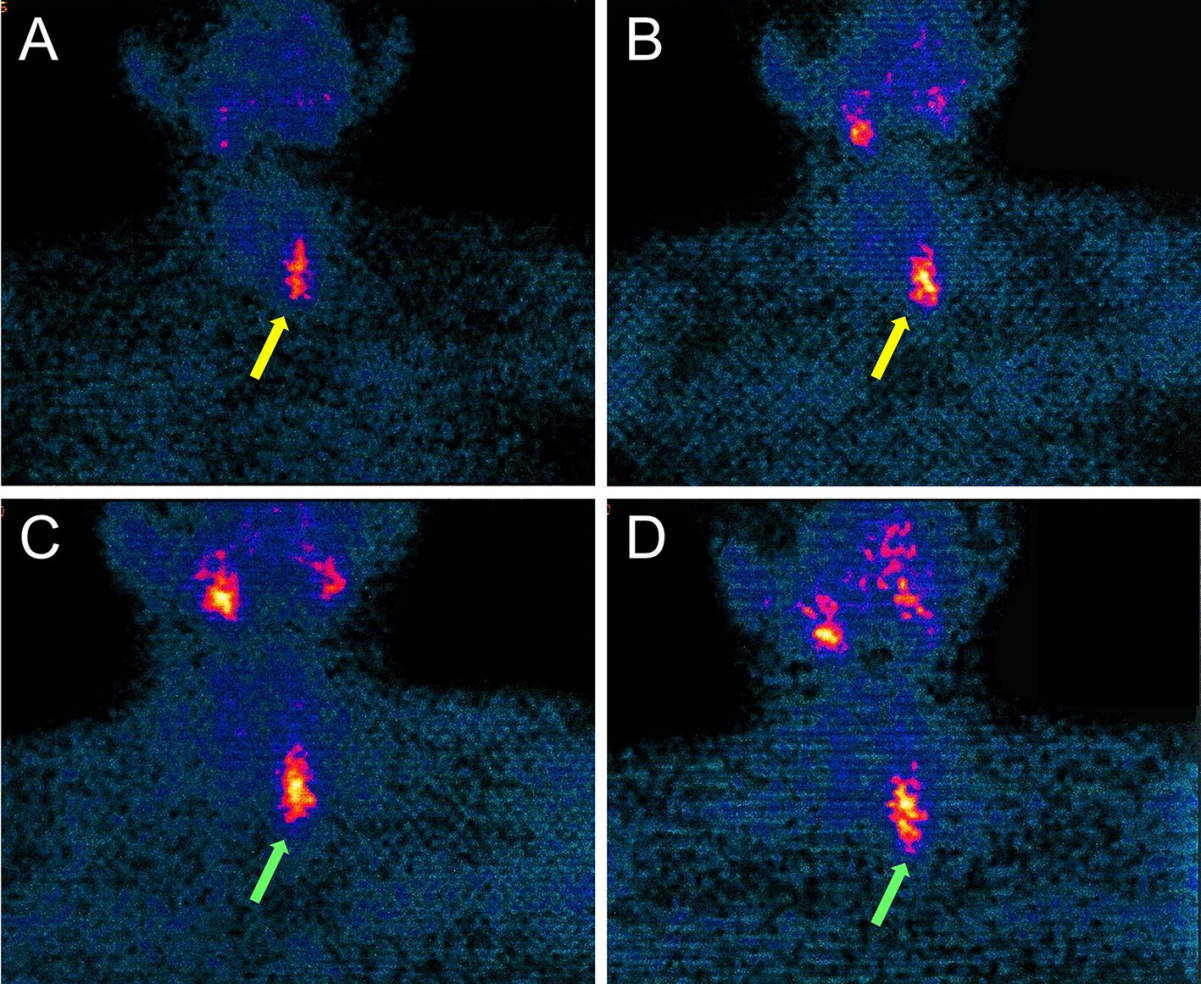
Early (**A**, **B**) and late (**C**, **D**) parathyroid SPECT imaging with [^99m^Tc]Tc-sestamibi suggesting the presence of hyperfunctioning parathyroid tissue in the lower left parathyroid lodge (yellow and green arrows)

The endocrinological consultations raised a diagnostic suspicion of primary hyperparathyroidism with ultrasound and color–power-Doppler evidence of hyperplastic parathyroid tissue in the lower left parathyroid lodge of 33.3 mm × 11.2 mm × 16.6 mm with contextual vascularization (Fig. 1). Parathyroid SPECT imaging was performed after intravenous administration of 296 MBq of [^99m^Tc]Tc-sestamibi and subsequent integration with 185 MBq of ^99m^Tc-perthecnetate. The images were obtained 15 min after tracer injection (early phase) and 180 min after tracer injection (late phase). Early phase showed a focal uptake in the lower left parathyroid lodge, while late phase did not show any wash-out confirming the diagnosis of hyperfunctioning parathyroid tissue in the lower left parathyroid lodge (Fig. 2).

The patient, treated with cinacalcet hydrochloride 30 mg 1 capsule twice daily, was referred to Department of Advanced Biomedical Sciences, University of Naples Federico II to perform [^18^F]fluorocholine PET/CT before surgical treatment according to EANM guidelines (Petranović Ovčariček et al. 2021) to evaluate the potential presence of ectopic and supernumerary glands in a patient with primary hyperparathyroidism. PET/CT unenhanced scan was acquired using a PET/CT Ingenuity TF (Philips Healthcare, Best, The Netherlands) 5 min and 60 min after administration of 280 MBq of [^18^F]fluorocholine (activity range 200–300 MBq according to body weight) as previously described (Nappi et al. 2022).

**Fig. 3:**
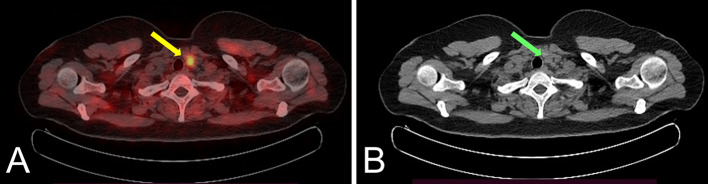
Axial [^18^F]fluorocholine PET/CT images (**A**) showing hyperfunctioning parathyroid in the middle-lower third of the left thyroid lobe (SUVmax 6.0 in the late phase) (yellow arrow) and CT coregistration (**B**) (green arrow)

**Fig. 4:**
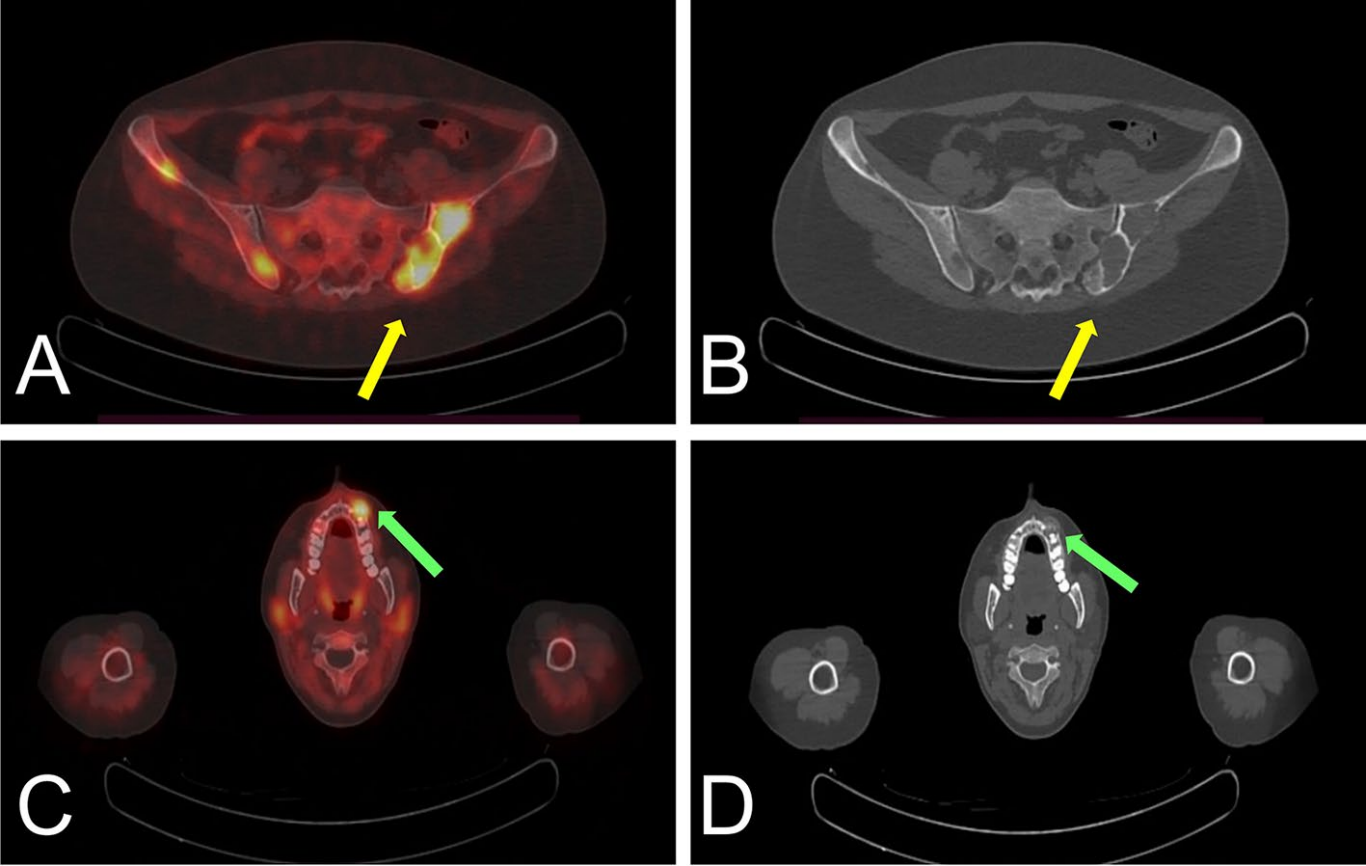
Axial [^18^F]fluorocholine PET/CT images (**A**) showing an avid lesion involving left iliac bone (SUVmax 7.9) and CT coregistration (**B**) (yellow arrows); axial [^18^F]fluorocholine PET/CT images (**C**) showing an avid lesion on left mandibular arch (SUVmax 5.6) and CT coregistration (**D**) (green arrows)

As illustrated in Fig. 3, [^18^F]fluorocholine PET/CT scan showed focal uptake of the tracer, both in early and late acquisitions, posteriorly to the lower third of the left thyroid lobe, confirming the diagnosis of hyperfunctioning parathyroid tissue demonstrating a maximum standardized uptake value (SUVmax) of 6.3 in the early phase and of 6.0 in the late phase. On the whole-body images, multiple areas of increased tracer uptake were observed on bone compartment, corresponding to skeletal lesions with morpho-structural alteration of the lytic type on co-registered CT. These bone images corresponded to: the left ethmoid labyrinth (SUVmax 7.2), left mandibular arch (SUV max 5.6), mandibular symphysis (SUVmax 7.3), right acromion (SUVmax 6.3), anterolateral side of the V and VII ribs of the right hemithorax (SUVmax 7.0), ileo-pubic branch bilaterally (SUVmax 4.5 on the left, SUVmax 7.9 on the right), the pubic symphysis (SUVmax 8.5 on the left, SUVmax 7.6 on the right), the right ischial bone (SUVmax 7.6) and the medial condyle of the left femur (SUVmax 2.4) (Fig. 4).

**Fig. 5:**
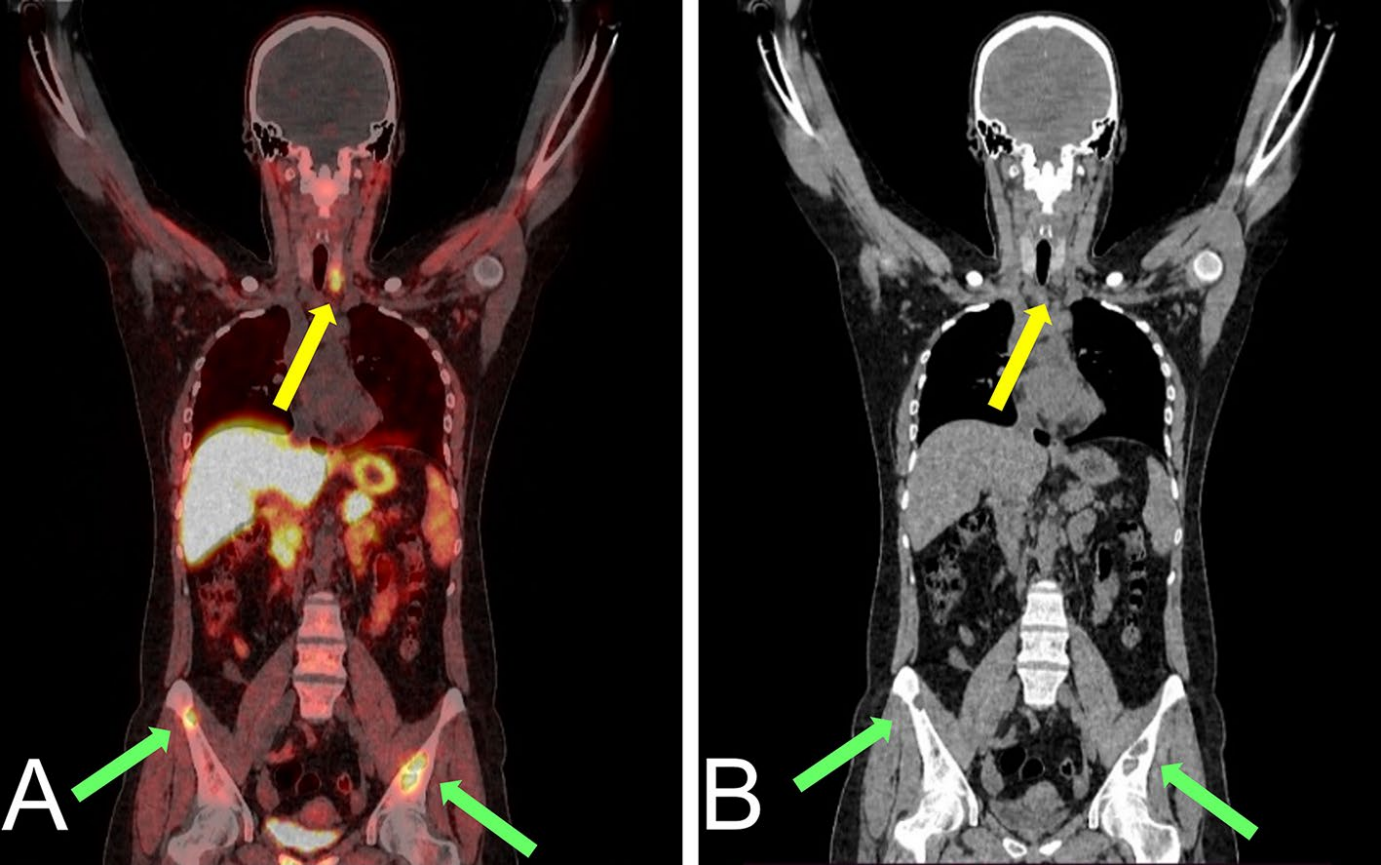
Coronal [^18^F]fluorocholine PET/CT images of hyperfunctioning parathyroid tissue (yellow arrows) on lower left parathyroid lodge and avid lesions involving iliac bone bilaterally (green arrows) (SUVmax 4.5 on the left and 7.9 on the right) (**A**) and CT coregistration (**B**)

**Fig. 6:**
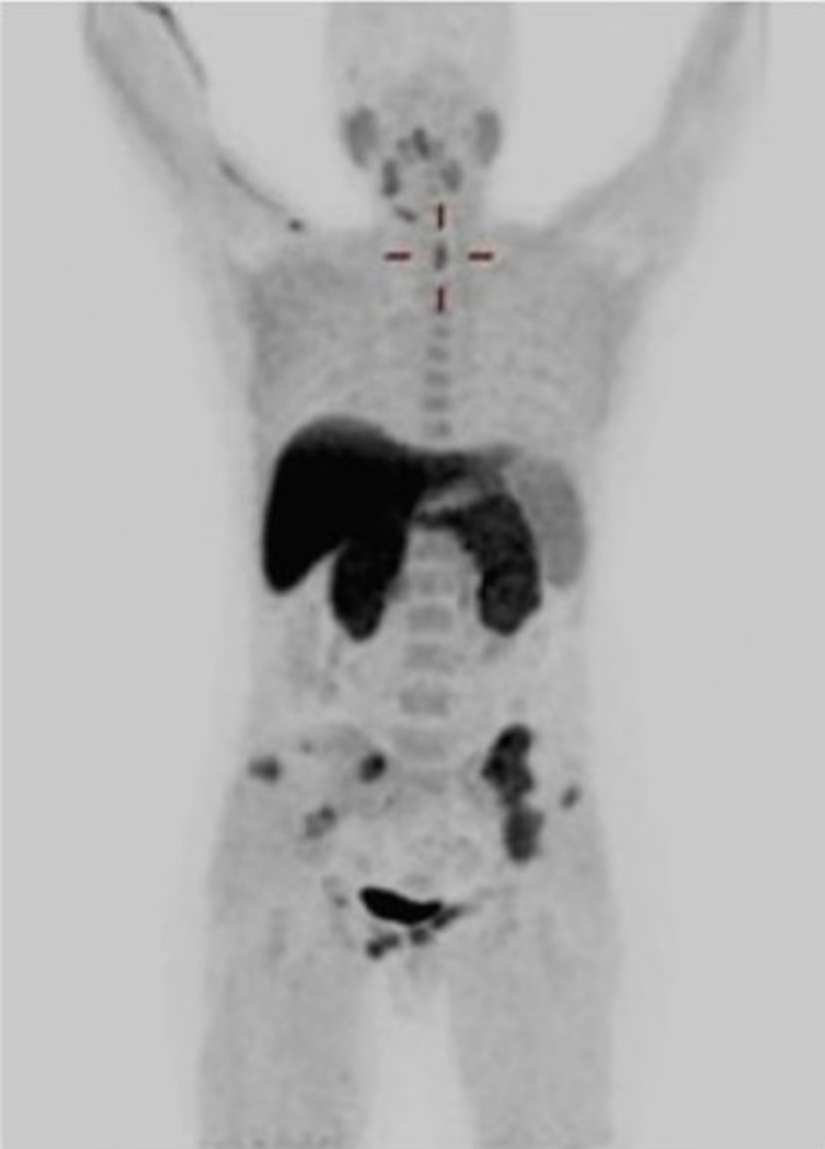
[^18^F]fluorocholine PET/CT maximum intensity projection

The final imaging report confirmed the clinical suspicion of hyperfunctioning parathyroid tissue on lower left parathyroid lodge and also reported the presence of multiple lytic bone lesions avid of [^18^F]fluorocholine suggesting hyperparathyroidism associated with skeletal involvement (Figs. 5 and 6). Subsequently, the patient underwent, after appropriate surgical consultation, a surgical removal of the lesion of the lower left parathyroid. Pathological examination of the surgical tissue confirmed the presence of hyperplastic parathyroid tissue compatible with parathyroid adenoma.

## Discussion

We report for the first time a case of a patient with evidence of both hyperfunctioning parathyroid tissue and multiple lytic bone lesions on [^18^F]fluorocholine PET/CT imaging. The association of multiple bone lesions with endocrine disorders has been widely investigated (Weinstein et al. 1991; Weinstein 2006; Chapurlat and Orcel 2008; Collins et al. 2012; Javaid et al. 2019; Hartley et al. 2019; Mazabraud et al. 1967). In particular, the relationship of multiple lesions due to fibrous dysplasia and endocrine disorders such as hyperparathyroidism has been described by McCune (1936) and Albright et al. (1937). However, to date no clinical cases showing high uptake of [^18^F]fluorocholine at PET/CT imaging, on hyperfunctioning parathyroid and on bone lytic lesions at the same time have been described.

Chapurlat and Orcel (2008) explained how bone fibrous dysplasia, characterized by bone pain, deformity and frequent fractures, is also associated with endocrine disorders and a specific genetic mutation. The genetic alteration is characterized by a missense mutation of the gene that codes for a subunit of the stimulatory G-protein, Gs, in the guanine nucleotide binding, alpha stimulating (GNAS) complex locus in chromosome 20q13. The activation of the GNAS gene determines high levels of cAMP that induces c-fos overexpression in signal cascade, resulting in the formation of abnormal bone tissue, in this case fibrous tissue (Weinstein et al. 1991; Weinstein 2006). In addition, a concomitant increase in osteoclasts leads to greater bone resorption and the formation of osteolytic lesions. Consequently, bone mineralization defects will also be present in these patients, which will result in biochemical alterations, such as increased PTH and hypovitaminosis D. As for the possible therapies of this rare pathology, the use of bisphosphonates and biological therapy, which will inhibit the altered gene path, appear to be promising, although for both therapeutic alternatives further research is needed (Chapurlat and Orcel 2008).

Turcu et al. (Turcu and Clarke 2013) in a retrospective review on 13,574 patients with primary hyperparathyroidism and 1272 cases with fibrous dysplasia identified only 10 patients diagnosed with both pathologies and some cases did not show any association with any genetic disorder. Thus, although the combination of both disorders is an unusual circumstance, physicians should be aware of the possibility to observe these two pathological patterns on [^18^F]fluorocholine PET/CT imaging.

## Conclusion

The present case report highlights the potential of whole-body [^18^F]fluorocholine on PET/CT to assess two different disease patterns in one imaging session and the possibility to identify a whole pathological picture of complex disorders, such as the presence of parathyroid adenoma and multiple bone lesions, in a single diagnostic setting.
